# Dyspnea in the Supine Position after Anterior Cervical Discectomy and Fusion

**DOI:** 10.1055/s-0038-1653979

**Published:** 2018-05-23

**Authors:** Hua Zhang

**Affiliations:** 1 Department of Orthopaedic Surgery, Zhejiang University, the Second Affiliated Hospital, School of Medicine, Hangzhou City, Zhejiang Province, China

**Keywords:** dyspnea, anterior cervical discectomy and fusion, pharyngeal, obstructive sleep apnea, soft tissues

## Abstract

Posterior occipitocervicothoracic fusion in a flexed position may cause dyspnea, and the onset of obstructive sleep apnea after anterior upper cervical fusion. However, there are no reports of dyspnea occurring after anterior lower cervical fusion. Here, we present an unusual case of dyspnea in the supine position after a C5-C6 anterior cervical discectomy and fusion.

Posterior occipitocervicothoracic fusion in a flexed position may cause dyspnea, 1 and the onset of obstructive sleep apnea (OSA) after anterior upper cervical fusion. 2 However, there are no reports of dyspnea occurring after anterior lower cervical fusion. Here, we present an unusual case of dyspnea in the supine position after a C5-C6 anterior cervical discectomy and fusion (ACDF).

## Case Report

A 60-year-old man presented with pain and numbness in his right arm for 1 month, which had worsened progressively. He had suffered from OSA for 7 years. His Epworth Sleepiness Scale 3 score was 8 points.

Physical examination revealed mild dysesthesia in the right forearm, thumb, and index finger. Slight motor weakness was detected in his right wrist extensors.

**Fig. 1:**
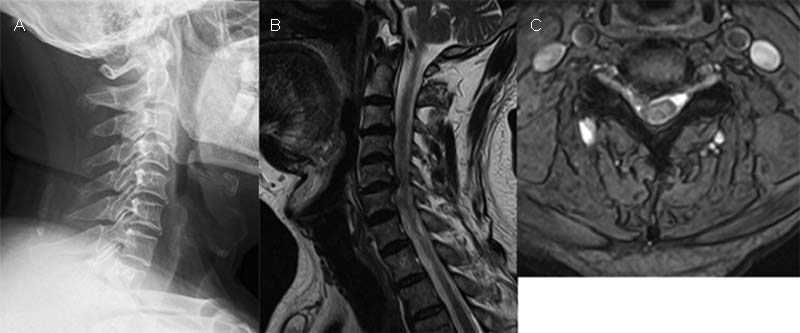
Preoperative images of the cervical spine: ( **A** ) X-rays, ( **B** ) sagittal magnetic resonance imaging (MRI), and ( **C** ) axial MRI at the C5/6 level.

Plain radiographs of the cervical spine revealed vertebral anterior osteophytes at C5/6 and C6/7, and no instability of the cervical spine ( Fig. 1A ). Magnetic resonance imaging indicated disc herniation at C5/6 toward the right side ( Fig. 1B and C ).

The patient underwent resection of the C5/6 disc and fusion using a titanium plate (Slim Loc Anterior Cervical *Plate* System, Johnson & Johnson Ltd.), screws, and a polyetheretherketone cage (Cervious, Johnson & Johnson Ltd.) with allogeneic bone grafts. During surgery, to remove the disc extrusion, we resect part of the posterior longitudinal ligament at the C5/6 disc level. The retraction used during surgery is a stainless steel manually held retractor with a width of ∼3 cm, and it placed under longus coli muscle. The operating time was ∼95 minutes, with an estimated blood loss of 30 mL. Intraoperatively, the volume of bleeding was ∼30 mL. Postoperatively, 80 mg of methylprednisolone (qd, for 3 days) was administered to prevent possible spinal cord edema. There was no plan to use postoperative intervention such as continuous positive airway pressure (CAPA) for OSA, because the patient did not feel so bad and get CAPA preoperation.

The next morning (postoperative day 1), the pain in the arm had disappeared and the numbness had improved. However, the patient complained that he could not breathe when in a supine position in awake state at the previous night. However, when he was in a lateral position or standing upright, the dyspnea improved markedly. No dysphagia was observed when he was allowed to drink water and eat porridge.

**Fig. 2:**
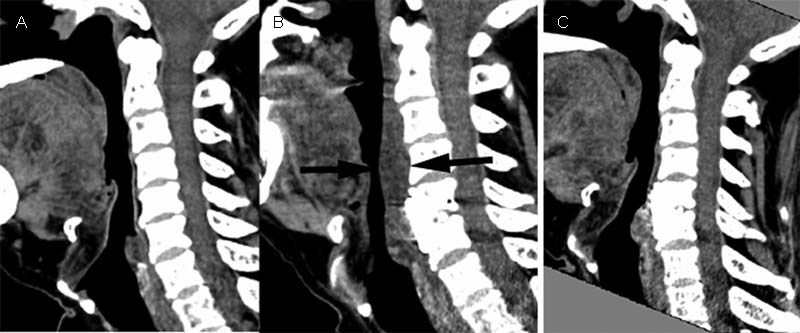
Sagittal reconstruction of cervical computed tomography images: ( **A** ) before, ( **B** ) 2 days, and ( **C** ) 2 months after the surgery. The middle pharyngeal space was reduced after the anterior cervical discectomy and fusion, leaving a thickened posterior pharyngeal wall and prevertebral soft tissues (between the black arrows in **B** ). The stenosis was improved at follow-up.

**Fig. 3:**
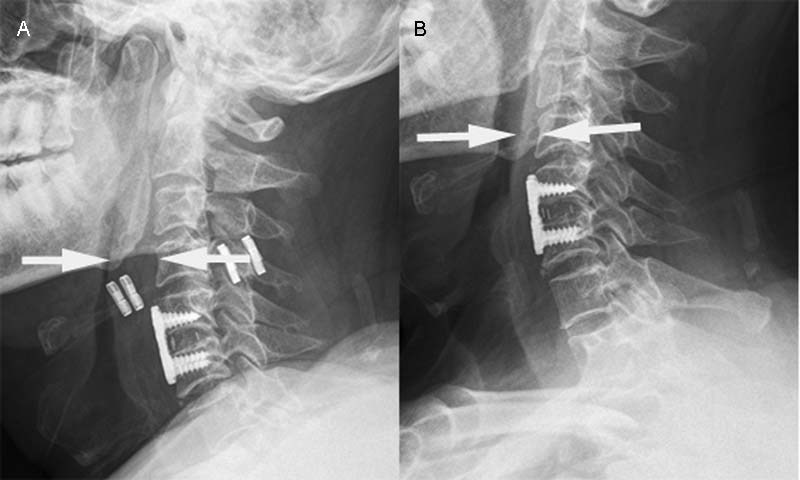
Postoperative X-rays of the cervical spine: ( **A** ) 2 and ( **B** ) 28 days after the surgery. The middle pharyngeal space was reduced leaving a thickened posterior pharyngeal wall and prevertebral soft tissues (between the white arrows in **A** ).

On postoperative day 2, X-rays and computed tomography (CT) of the cervical spine were performed routinely to check the location of the plate. CT showed a reduction in the middle pharyngeal space, with thickening of the posterior pharyngeal wall and prevertebral soft tissues from C2 to C6 ( Fig. 2B ). A lateral X-ray of the cervical spine also revealed a marked reduction in the middle pharyngeal space ( Fig. 3A ).

The thickness of the prevertebral soft tissues exceeded that of the plate. Measuring the thickness of the soft tissues between the posterior border of the pharyngeal space and the anterior vertebral wall on CT at the C4 level in the middle section, the preoperative thickness was 2 mm, whereas the postoperative thickness was 14 mm, which exceeded the thickness of the plate (2 mm) ( Fig. 2 ).

The patient was not treated for the dyspnea in a supine position. However, he had to sleep in a lateral position to prevent dyspnea. Twenty-eight days after the surgery, the dyspnea in a supine position in awake state had improved markedly and X-rays showed that the pharyngeal stenosis had disappeared ( Fig. 3B ). At the 2-month follow-up, the patient did not complain of dyspnea in a supine position, and CT showed that the thickening of the posterior pharyngeal wall and prevertebral soft tissues from C2 to C6 had disappeared ( Fig. 2C ). However, the patient still had OSA postoperation, and the Epworth Sleepiness Scale was 8 points.

## Discussion

Anterior cervical fusion is often used to treat patients with radiculopathy and myelopathy after the failure of nonoperative treatment. In the two reports on upper airway obstruction after anterior cervical fusion in English, the metal plate used for the cervical fusion was the cause of the upper airway obstruction. 2 4 We were not sure that this was applicable to lower cervical fusions because the thyroid cartilage and cricoid cartilage support the upper airway below the C5 level. The metal plate caused anterior displacement of the posterior pharyngeal wall, affecting the airflow by narrowing the middle pharyngeal space of the upper airway. 2 4 In our case, however, the airway obstruction improved when the thickening of the posterior pharyngeal wall and prevertebral soft tissues from C2 to C5 disappeared. It strongly supports the effect of the anterior cervical soft tissue swelling on the patient's dyspnea.

Kanti and Aparna 4 reported a case of difficulty breathing during sleep after cervical fixation with a plate and screws from C3 to C5. A lateral cephalogram showed that the upper top of the fixation device impinged on the patient's middle pharyngeal space, resulting in obstruction of the upper airway. In addition, the patient's Mallampati score was IV. The higher the score from I to IV, the more likely that the patient has OSA. 5 Their patient could not tolerate a continuous positive airway pressure (CPAP) device and chose a Thornton Adjustable Positioner device because it is effective in the treatment of mild-to-moderate OSA. It reduces the associated health risks without surgical intervention, CPAP, or medication.

The other study reviewed 12 patients who developed OSA syndrome after anterior cervical spine fusion. 2 Four subsequent patients were studied prospectively before C2 to C4 anterior fusion and documented to have OSA based on a questionnaire, visual analogue scales, polysomnography, and multiple sleep latency tests. The authors found that placement of the anterior fixation device reduced the size of the middle pharyngeal space. However, these cases differed from our case and their prime cause was considered to be the anterior cervical fixation.

We could not find any English-language reports of cases of prolonged upper-airway obstruction after C5/6 ACDF. Note that the fusion range in our case was lower than in the published cases. Considering the soft-tissue damage caused by the retractor during an anterior cervical operation, the thickening of the posterior pharyngeal wall and prevertebral soft tissues from C2 to C5 may have reduced the middle pharyngeal space, causing pharyngeal stenosis. In addition, intubation may cause pharyngeal injury and edema because it is difficult to intubate some patients. 1 6 Figs. 2 and 3 show the thickness of the soft tissues in our patient and demonstrate the much greater thickness at C4 2 days after surgery compared with that preoperatively and at follow-up. This suggests that the thickening of the posterior pharyngeal wall and prevertebral soft tissues from C2 to C5 played a role in the dyspnea.

Sleep apnea is a multifactorial disease with many identified risk factors and mechanisms. The cervical spine can contribute to this disease. Specific cervical column pathologies that cause sleep apnea, such as osteophytes, osteochondromas, abnormal morphology, and rheumatoid arthritis with occipitocervical lesions, have been identified in case reports. 7 Some studies have investigated the association between the metal plate used in anterior cervical fusion surgery and postoperative sleep apnea. 2 4 To our knowledge, however, there are no reports on an association between anterior cervical soft-tissue swelling and postoperative dyspnea. However, a significant increase in anterior cervical soft-tissue swelling after ACDF has been reported. 8 Dysphagia is common in the early postoperative period after ACDF, and may be related to soft-tissue swelling, which has been implicated as a cause of postoperative dysphagia. 9 10 Our patient had suffered from OSA for 7 years and his Epworth Sleepiness Scale score was 8 points, which may have contributed to the postoperative dyspnea. In a supine position, the hypertrophic tongue falls posteriorly, causing further pharyngeal space stenosis, which may be one of the risk factors for dyspnea. In contrast, in a lateral position or sitting, the dyspnea was markedly better.
